# Disrupted superior collicular activity may reveal cervical dystonia disease pathomechanisms

**DOI:** 10.1038/s41598-017-17074-x

**Published:** 2017-12-01

**Authors:** Eavan M. Mc Govern, Owen Killian, Shruti Narasimham, Brendan Quinlivan, John B. Butler, Rebecca Beck, Ines Beiser, Laura W. Williams, Ronan P. Killeen, Michael Farrell, Sean O’Riordan, Richard B. Reilly, Michael Hutchinson

**Affiliations:** 1 Department of Neurology, St Vincent’s University Hospital Dublin, Dublin, Ireland; 2 School of Medicine & Medical Science, University College Dublin, Dublin, Ireland; 3 Trinity Centre for Bioengineering, Trinity College, The University of Dublin, Dublin, Ireland; 4 School of Engineering, Trinity College, The University of Dublin, Dublin, Ireland; 5 School of Medicine, Trinity College, The University of Dublin, Dublin, Ireland; 6 School of Mathematical Sciences, Dublin Institute of Technology, Kevin St, Dublin, Ireland; 7 Department of Neuroradiology, St. Vincent’s University Hospital, Elm park, Dublin, Ireland; 8 Department of Neuropathology, Beaumont Hospital, Dublin, Ireland

## Abstract

Cervical dystonia is a common neurological movement disorder characterised by muscle contractions causing abnormal movements and postures affecting the head and neck. The neural networks underpinning this condition are incompletely understood. While animal models suggest a role for the superior colliculus in its pathophysiology, this link has yet to be established in humans. The present experiment was designed to test the hypothesis that disrupted superior collicular processing is evident in affected patients and in relatives harbouring a disease-specific endophenotype (abnormal temporal discrimination). The study participants were 16 cervical dystonia patients, 16 unaffected first-degree relatives with abnormal temporal discrimination, 16 unaffected first-degree relatives with normal temporal discrimination and 16 healthy controls. The response of participant’s superior colliculi to looming stimuli was assessed by functional magnetic resonance imaging. Cervical dystonia patients and relatives with abnormal temporal discrimination demonstrated (i) significantly reduced superior collicular activation for whole brain and region of interest analysis; (ii) a statistically significant negative correlation between temporal discrimination threshold and superior collicular peak values. Our results support the hypothesis that disrupted superior collicular processing is involved in the pathogenesis of cervical dystonia. These findings, which align with animal models of cervical dystonia, shed new light on pathomechanisms in humans.

## Introduction

Cervical dystonia is a hyperkinetic movement disorder characterised by sustained or intermittent muscle contractions causing abnormal movements and postures^1^. It is the most common phenotype of adult onset focal isolated dystonia (AOIFD), of which several phenotypes exist. The pathogenesis and the neural networks underpinning this condition remain unknown. While cervical dystonia is considered to be essentially due to basal ganglia dysfunction^1–6^, mounting evidence indicates a wider network disorder involving cortical, subcortical and cerebellar regions^7–9^. Understanding how these various structures interact with the basal ganglia is important when considering possible pathomechanisms in cervical dystonia^10–12^. Subcortical structures including the midbrain interstitial nucleus of Cajal, midbrain neural integrators and the superior colliculus form part of this network and have been implicated in its pathogenesis^13–17^. Alterations in basal ganglia-brainstem and cerebellum-brainstem connections are also considered important^18–21^. Disruption of inhibitory input to the superior colliculus in macaques results in a primate model of cervical dystonia^17,22^. While deficient inhibition has long been considered important in the pathogenesis of dystonia^23^, how the superior colliculus fits into this paradigm has yet to be studied in humans.

Cervical dystonia is postulated to be a poorly penetrant autosomal dominant condition^18–20^. However causative genes have been identified in <1% of cases^15–17^. This shortfall in gene discovery has stimulated a search for endophenotypes and alternative pathomechanistic theories^22^. Aberrant sensorimotor plasticity has been linked to the pathogenesis of dystonia^8,23,24^, in particular focal-hand dystonia, a phenotype of AOIFD frequently associated with overuse^24–28^; cervical dystonia has been less well studied^29^. The concept of sensory dysfunction as being primary to the development of cervical dystonia is supported by the co-existence of sensory symptoms including geste-anatagoniste^30–32^. Tactile and visual perception in dystonia have been studied using the temporal discrimination threshold (TDT), which measures the ability to perceive two sequential sensory stimuli as being temporally separate. An abnormal TDT has emerged as a reliable mediational endophenotype for cervical dystonia and, as such, may help to uncover pathomechanims in cervical dystonia^22–24^. This marker is abnormal in 97% of patients with cervical dystonia with a specificity of 98–100%^23,25–27^ and demonstrates autosomal dominant transmission in families of patients with sporadic and familial cervical dystonia^25–27^. Unaffected relatives and patients were selected in the present study to investigate the role of superior collicular dysfunction in cervical dystonia. Unaffected relatives with abnormal temporal discrimination (up to 50% of female relatives & 20% male relatives^33^) are hypothesised to be non-manifesting gene carriers. This group may thus manifest similar alterations in brain activity to patients (without the complication of secondary effects due to motor manifestations). These unaffected relatives form an interesting study group because, by studying them one may examine disordered brain activation resulting from the endophenotype alone (an abnormal TDT) without the secondary effects from phenotype manifestation (cervical dystonia).

Covert attentional orienting detects change in the environment and alerts the individual to a salient stimulus; the superior colliculus is a key node involved in this process^34,35^. Detection of environmental change confers survival advantage amongst species and relies on the accurate detection of approaching (looming) objects^36–38^. Looming-sensitive neurons have been identified in the superficial layer of the optic tectum in early vertebrates and in the superior colliculus in mammals^39,40^. In man, a functional magnetic resonance imaging (fMRI) study revealed that looming stimuli (but not random stimuli) activated the superior colliculus^41^.

To assess the relationship between temporal discrimination, superior colliculus activity and cervical dystonia, the aim of this study was to examine, by fMRI, the activation of the superior colliculi in response to looming stimuli in cervical dystonia patients, their unaffected relatives with normal and abnormal temporal discrimination and healthy controls.

## Results

### Temporal discrimination threshold testing

**Figure 1:**
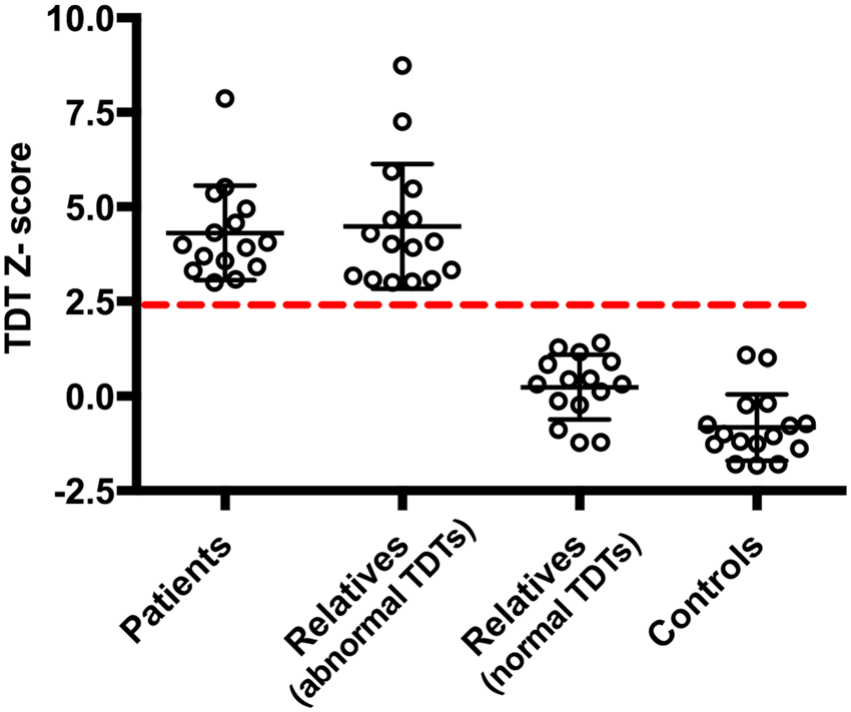
Participants’ temporal discrimination threshold Z-scores: Temporal discrimination threshold (TDT) Z-scores in 16 patients, 32 relatives and 16 healthy controls. The black open circles represent individual TDT Z-scores. An abnormal TDT Z-score was defined ≥2.5 standard deviations above the age- and sex- matched population mean. The dashed-red line denotes a TDT Z-score of 2.5. All sixteen cervical dystonia patients had TDT Z-scores ≥ 2.5, 16 unaffected relatives had a TDT Z-score ≥ 2.5 (relatives with abnormal TDT), 16 unaffected relatives had a TDT Z-score ≤ 2.5 (relatives with normal TDT) and all 16 healthy controls had a TDT Z-score ≤ 2.5.

Temporal discrimination threshold testing results from sixty-four participants were expressed as Z-scores (Fig. 1). All sixteen cervical dystonia patients had abnormal TDTs (mean Z-score 4.3; SD ± 1.2). Sixteen of the thirty-two first degree relatives had abnormal TDTs (mean Z-score 4.5; SD ± 1.6) and sixteen had normal TDTs (mean Z-score 0.25; SD ± 0.82). All sixteen healthy control subjects had normal TDTs (mean Z-scores – 0.64; SD ± 1.02).

### Functional Magnetic Resonance Imaging study

#### Behavioural analysis

There was no statistical difference in responses times between the three conditions: looming stimuli, receding stimuli and random stimuli.

### Whole brain analysis

**Figure 2:**
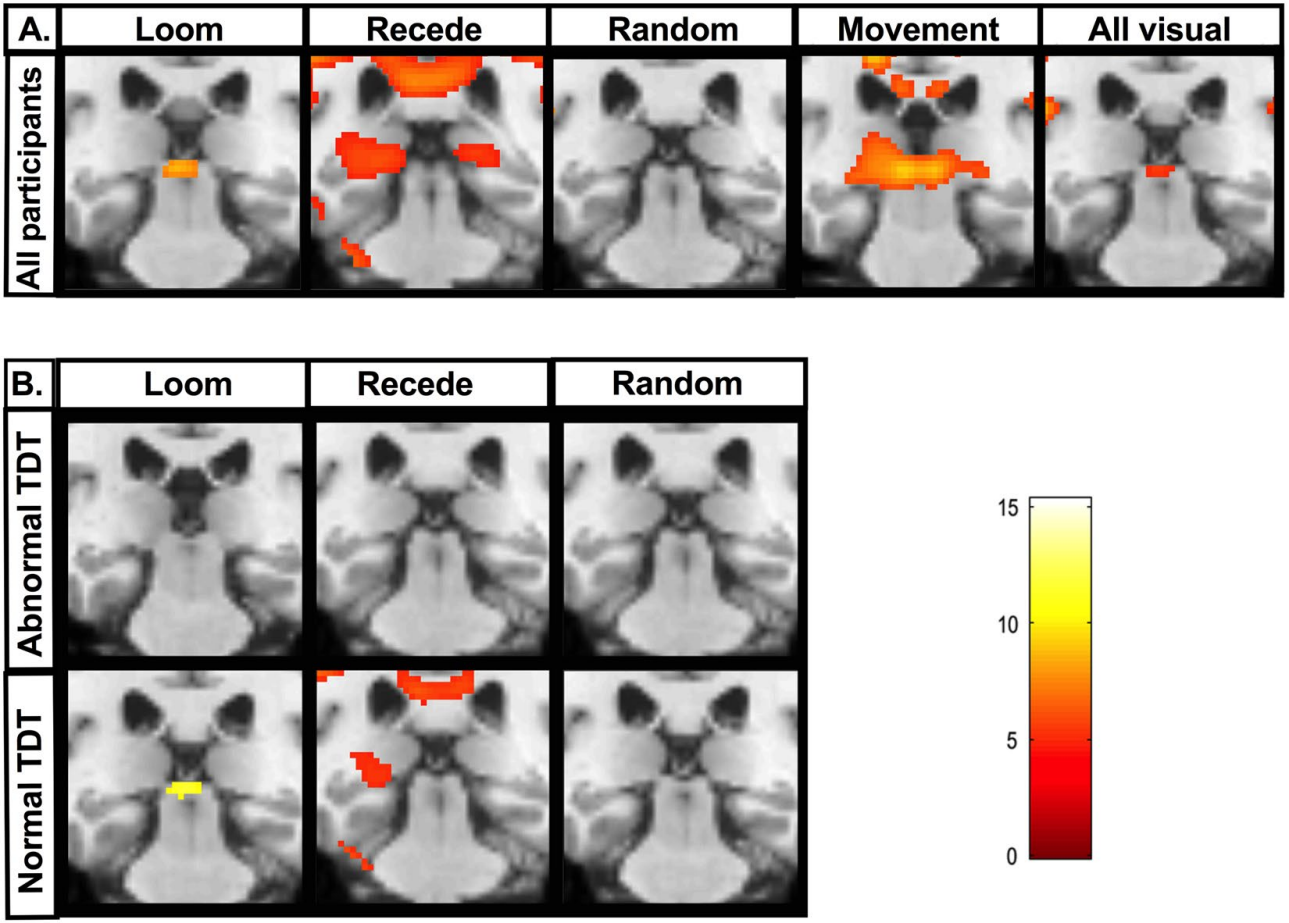
Whole brain analysis of superior collicular activation to visual stimuli: (**A**) Whole brain, whole group 2^nd^ level general linear model (GLM) analysis in all 64 participants (16 patients, 32 unaffected relatives, 16 healthy controls) for the following basic contrasts: loom, recede, random, structured movement (loom & recede) and all visual (loom, recede & random). Coronal brain images are displayed. Slices were selected to highlight peak superior collicular activity. A Family wise error corrected p-value < 0.05 is used. K = cluster size. A significant and focal activation of both superior colliculi is seen for the loom condition (K = 69, p < 0.002). The recede random and structured movement conditions failed to demonstrate statistically significant clusters at superior collicular level. (**B**) Segmented group 2^nd^ level GLM analysis brain analysis in 32 participants with abnormal temporal discrimination thresholds (TDTs) (16 cervical dystonia patients and 16 relatives with abnormal TDTs) and 32 participants with normal TDTs (16 relatives and 16 control participants with normal TDTs). A statistically significant cluster at superior collicular level is observed for the looming condition in the normal TDT group. In the abnormal TDT group, no statistically significant clusters were observed within the superior colliculus boundary for the loom, recede or random condition.

Second level general linear model (GLM) analysis was carried out to include all 64 participants. Whole group analysis for the basic contrast 1-sample t-test (loom >/< 0, recede >/< 0 & random >/< 0) are presented (Fig. 2A). The loom condition demonstrated a significant and focal activation of both superior colliculi. The recede and random conditions failed to demonstrate statistically significant activations at superior collicular level. The activation in response to structured movement (loom & recede) was less focal than in the loom condition. The images for all visual input (loom, recede and random) showed a weaker activation pattern than that observed for structured movement. The 2-sample t-test contrast demonstrated a statistically significant cluster for the loom > random condition at both superior colliculi. No statistically significant clusters were observed within the superior colliculus boundary for the recede > random or the loom > recede condition. Segmented group analysis for the 1-sample and 2-sample t-test contrast is presented (Fig. 2B). A statistically significant cluster at superior collicular level is observed for both the looming condition and the loom > random condition in the normal TDT group. No such activations are observed for the abnormal TDT group. The recede and random conditions failed to demonstrate statistically significant clusters in either group at superior collicular level.

**Figure 3:**
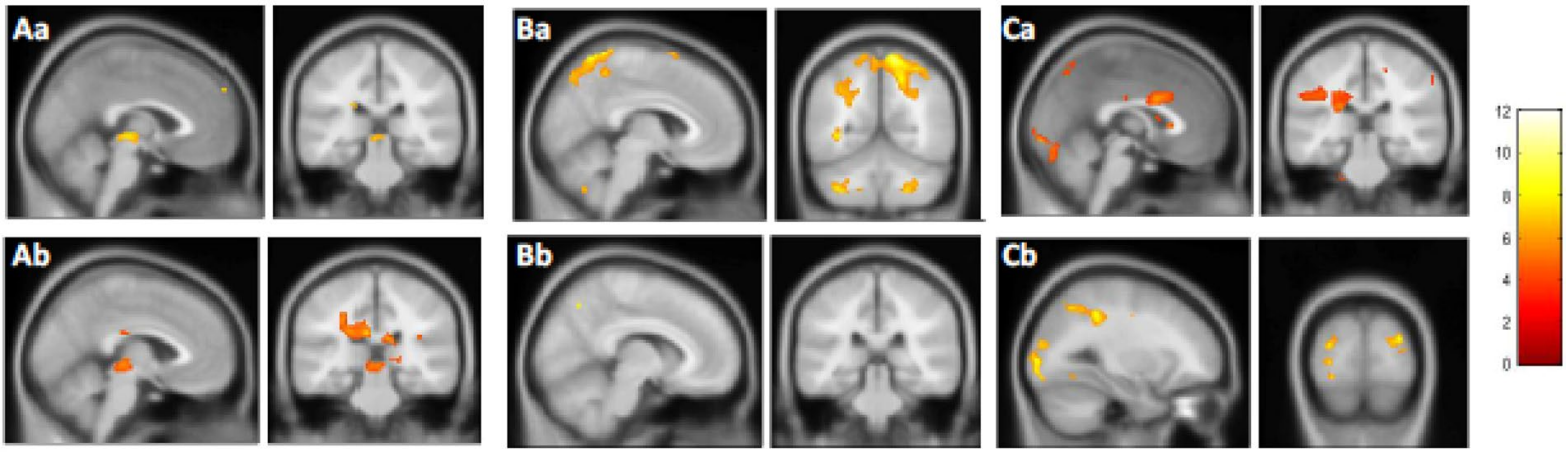
Whole brain, whole group analysis for the three different contrasts. Whole brain, whole group 2^nd^ level general linear model (GLM) analysis in 32 participants with normal temporal discrimination threshold (TDT) and 32 participants with abnormal TDT for the following three contrasts are presented; Aa = Loom > Random (normal TDT); Ab = Loom > Random (abnormal TDT); Ba = Loom > Recede (normal TDT); Bb = Loom > Recede (abnormal TDT); Ca = Recede > Random (normal TDT); Recede > Random (abnormal TDT). A Family wise error corrected p-value < 0.05 is used. TDT = temporal discrimination threshold. K = cluster size. BA = Broadmann’s area. Statistically significant clusters were observed in the following areas for each of the contrasts: Aa = Left BA19 (K = 1151, p < 0.000), Right visual association cortex (K- 757, p < 0.001), Right superior colliculus (K=299, p < 0.05). Ab = Left fusiform gyrus (K = 5374, p < 0.000), Right BA19 (K = 2122, p < 0.000), Right thalamus (K = 312, p < 0.018). Ba = Right BA7 (K = 5196, p < 0.000), Left occipital cortex (K = 372, p < 0.038) Left visual association cortex (K = 372, p < 0.038). Bb = Left sensory association area (K = 27, p < 0.001), Right BA39 (K = 95, p < 0.000), Right BA7 (K = 27, p < 0.001). Ca = Right visual association cortex (K = 16078, p < 0.000), Cb = Right visual association cortex (K = 427, p < 0.000).

Whole group analysis for loom > random, recede > random and recede > random contrasts are presented for the 32 participants with normal TDT (unaffected relatives and healthy controls) and the abnormal TDT group (patients and unaffected relatives) (Fig. 3). A statistically significant cluster at superior collicular level is observed for the loom > random contrast (K = 299, p < 0.05) in the normal TDT group. This activation is not observed in the abnormal TDT group.

### Region of interest analysis

#### Effect of condition

**Figure 4:**
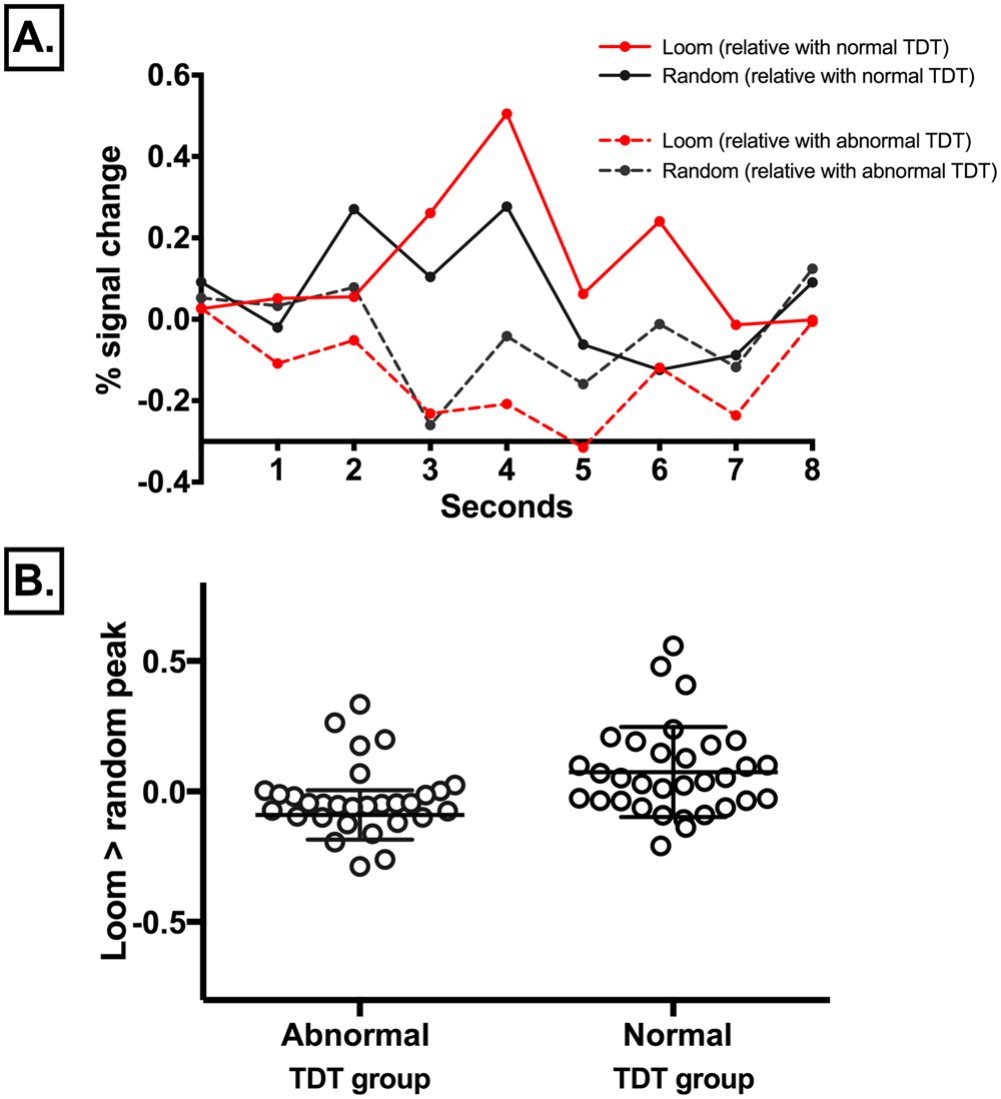
Event related time courses: (**A**) Event-related time course for the superior colliculus during the loom and random condition in two candidate subjects: one relative with abnormal temporal discrimination threshold (TDT) and one relative normal TDT. Red solid line = looming condition in a relative with normal TDT; black solid line = looming condition in a relative with normal TDT; red dashed line = random condition in a relative with abnormal TDT; black dashed line = random condition in a relative with abnormal TDT. In the relative with normal TDT, the peak percent signal change is observed at 4 seconds for the loom condition. This peak percent signal change for the loom condition was reversed in the relative with an abnormal TDT. (**B**) Loom > random peak signal in all 64 participants: 32 participants with abnormal TDT (patients and unaffected relatives) and 32 participants with normal TDT (unaffected relatives and healthy controls). Each circle represents an individual’s peak signal for the loom > random contrast. A significant difference was observed in the peak signal for the loom > random contrast between the abnormal TDT group (M = −0.092, SD = 0.266) and the normal TDT group [M = 0.076, SD = 0.17; t(52) = 3.0, p = 0.004]. The magnitude of the differences in the means was very large (eta squared = 0.14).

The raw event-related time courses were extracted for loom, recede and random conditions from each participant’s superior colliculus. The greatest signal difference was observed between the loom and random condition (Fig. 4A). The peak percent signal change during the loom condition was observed at 4 seconds across participants with normal temporal discrimination. This peak percent signal change for the loom condition was absent in participants with abnormal TDTs. An independent-samples *t*-test was conducted to compare BOLD activations for the three main contrasts (loom > random, recede > random, loom > recede) for both the abnormal TDT and normal TDT group. A significant difference was observed for the loom > random contrast for the abnormal TDT group (M = −0.092, SD = 0.266) and the normal TDT group [M = 0.076, SD = 0.17; t (62) = 3.0, p = 0.004]. The magnitude of the differences in the means was very large (eta squared = 0.14) (Fig. 4B).

A less significant difference was observed for the recede > random contrast for the abnormal TDT group (M = −0.12, SD = 0.32) and the normal TDT group [M = 0.02, SD = 0.19; t(62) = 2.05, p = 0.05]. The magnitude of the differences in the means was moderate (eta squared = 0.06). There was no significant difference observed for the loom > recede contrast for the abnormal TDT group (M = 0.027, SD = 0.17) and the normal TDT group [M = 0.05, SD 0.27; t (62) = 0.08, p = 0.768]. Participants were then divided into four groups (Group 1: patients; Group 2: relatives with abnormal TDT; Group 3: relatives with normal TDT; Group 4: Healthy controls). A one-way between-groups analysis of variance (ANOVA) was conducted to explore the impact of group level on loom > random peak value. There was a statistically significant difference at the p < 0.05 in loom > random peak values for the four groups [F (3,60) = 5.85, p < 0.001]. The difference in the mean scores was large. The effect size, calculated using eta squared, was 0.23. Post-hoc comparisons using the Turkey Honest Significant Difference test indicated that the most statistically significant group difference was observed between relatives with abnormal TDTs (M = −0.18, SD 0.33) and relatives with normal TDTs (M = 0.12, SD = 0.20, p < 0.001).

#### Correlation analysis with temporal discrimination threshold

**Figure 5:**
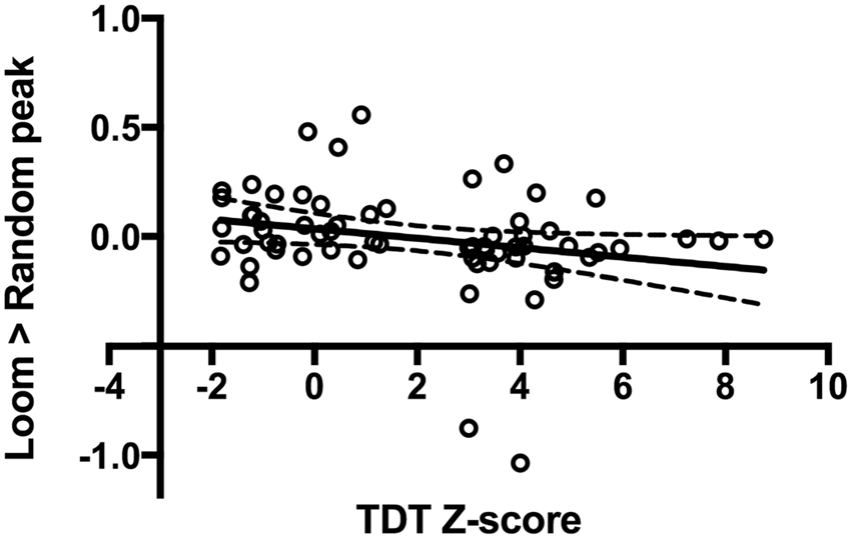
Correlation analysis: Pearson’s product moment correlation-coefficient analysis examining the relationship between superior collicular peak percent signal change for the loom > random contrast and temporal discrimination threshold (TDT) Z-score. Each open black circle represents one of the 64 participants; TDT Z-scores are plotted on the x-axis; the peak percent signal change observed for the loom > random contrast is plotted on the y-axis. The analysis shows a statistically significant negative correlation between the loom > random contrast and TDT Z-score [r = − 0.25, n = 62, p < 0.04], with lower levels of superior collicular activation associated with a higher (abnormal) TDT Z-score.

The relationship between superior collicular BOLD activation for each contrast (as measured by the peak percent signal) and TDT Z-score was investigated using Pearson Product-moment correlation-coefficient. Preliminary analysis was performed to ensure no violation of the assumption of normality, linearity and homoscedasticity. There was a statistically significant negative correlation between the loom > random contrast and TDT Z-score [r = −0.25, n = 62, p < 0.04], with lower levels of superior collicular activation associated with a higher (abnormal) TDT Z-score (Fig. 5).

There was a non-significant negative correlation between the recede > random contrast and TDT Z-score [r = −0.22, n = 62, p < 0.08]. There was a lack of association observed between the loom > recede contrast and TDT Z-score [r = 0.006, n = 62, p < 0.96].

## Discussion

Our results reveal that patients with cervical dystonia and their unaffected relatives with abnormal temporal discrimination, demonstrated (i) disrupted superior collicular activation; (ii) significantly reduced superior collicular activation for whole brain and region of interest analysis; and (iii) a statistically significant negative correlation between TDT Z-score and superior collicular peak values. These findings which provide evidence of a functional abnormality within the superior colliculus in both patients and first-degree relatives carrying a disease-specific endophenotype supports the hypothesis that dysfunctional superior collicular processing may be involved in the pathogenesis of cervical dystonia.

Our experimental paradigm was constructed principally to activate the superficial layer of the superior colliculus during the loom condition^41^. During whole-brain, whole-group analysis, we observed a significant and focal activation of the superior colliculus for the loom condition, whereas the recede and random conditions failed to induce significant activation within the superior colliculus boundary. The random condition produced the least superior collicular activation and the loom > random contrast revealed the most statistically significant cluster at superior collicular level. This contrast maximised superior collicular activation and controlled for inter-subject variability across participants. Our initial findings confirmed that our experimental paradigm was a robust method for producing superior collicular activation and aligned with the existing literature regarding optimal superior collicular activation^39,40^. Following our initial results, we proceeded to examine for any group differences observed during whole-brain analysis.

Whole-brain analysis revealed a between-group difference in superior collicular activation for the loom condition. Participants with abnormal temporal discrimination (cervical dystonia patients and relatives) had an absence of superior collicular activation to looming stimuli. There was no statistically significant difference amongst the cervical dystonia patients between the loom, recede and random conditions. In contrast, participants with normal temporal discrimination (relatives and controls) showed a significant activation to looming stimuli at superior collicular level. The absence of a normal activation pattern observed in participants with abnormal temporal discrimination (the endophenotype) suggests that they have disrupted superior collicular processing. To explore this, further, we proceeded to region-of-interest analysis, specifically focusing on functional activations at superior collicular level.

To improve the accuracy of region-of-interest analysis, we defined our superior collicular boundaries using anatomical landmarks determined at neuropathological dissection. Despite recent developments in automated anatomical labelling for cortical structures^42^, and detailed probabilistic atlases of macroscopic anatomy^43,44^, anatomical definition at individual level remains the optimal approach for ROI definition of small subcortical structures like the superior colliculus^45^.

Region-of-interest analysis revealed a statistically significant between-group difference in superior collicular activation. Patients and relatives with abnormal temporal discrimination had statistically significantly reduced superior collicular activation for the loom condition compared to participants with normal temporal discrimination. The greatest between-group difference in superior collicular activation was observed when comparing relatives with abnormal temporal discrimination and relatives with normal temporal discrimination. This between-group difference noted in superior collicular activation was further supported by the raw event-related time series extracted from each individual participant’s region-of-interest. In this analysis, the extracted peak signal from an individual’s superior colliculus should occur at four seconds. In participants with normal temporal discrimination a four second peak signal was observed for the loom condition. However, in those with abnormal temporal discrimination there was a reverse of this peak signal for the loom condition at four seconds (Fig. 4A).

Amongst cervical dystonia patients, there was no significant difference in either whole brain or region of interest analysis between those receiving regular botulinum toxin (BoNT) injections (n = 13) and those who did not (n = 3). Previous functional imaging studies have demonstrated a partial restoration of abnormal brain activations following BoNT injections^46–49^. Unlike our study, these protocols were devised to specifically examine the effect of BoNT on abnormal brain activations and imaging was frequently performed before and after injections. In our study repeat imaging was not performed and intervals following the last BoNT injection were variable. As such it was unlikely that any differences would exist between the two groups.

The findings from whole brain and region-of-interest analysis were corroborated further by correlation analysis. This analysis demonstrated a statistically significant negative correlation between superior collicular peak activations and individual TDT Z-scores, indicating that as superior collicular activation diminished, TDT Z-score increased (or worsened). The above findings suggest that superior collicular dysfunction may be involved in the disease-specific endophenotype (abnormal temporal discrimination). In accordance with the mediational endophenotype model which implies that one cannot acquire the disease without first having the endophenotype^50^, the superior colliculus is thus likely to play an important role in the pathogenesis of cervical dystonia.

The superior colliculus is one of the most GABAergic brain regions^51^ and the superficial layer is principally composed of GABAergic neurons.

Under normal conditions, neurons in the superficial layer of the superior colliculus respond to looming stimuli^39,40^; the time of the peak response in these neurons is linearly related to the size/speed ratio of the approaching object^52^. In our study, participants with normal temporal discrimination had this predicted response - a statistically significant greater activation to looming stimuli (loom > random contrast) at superior collicular level. Conversely, participants with abnormal temporal discrimination failed to demonstrate such activations. In this group - cervical dystonia patients and relatives with abnormal temporal discrimination - there was no significant superior collicular activation to looming stimuli. Thus, disrupted superior collicular processing is unique to those with an abnormal TDT (i.e. those with the endophenotype) and is *independent of phenotype* (cervical dystonia or unaffected relatives). This suggests that processing intrinsic to the superior colliculus is involved in the process of temporal discrimination.

Any reduction in GABA activity will not only have functional consequences for this dorsal midbrain structure but also its connections. The visuosensory neurons in the superficial layer of the superior colliculus exert inhibitory influences on the pre-motor neurons in the intermediate and deep layer of the superior colliculus^53,54^. The deep layer in turn projects via the tecto-reticulospinal and tectospinal pathways to the upper cervical spinal cord^55–58^. Prolonged duration firing of visuosensory neurons because of impaired GABA inhibition would cause hyperexcitability of the pre-motor neurons in the deep layer of the superior colliculus. These hyperexcitable premotor neurons could stimulate motor neurons in the upper cervical spinal cord perhaps resulting in the abnormal, jerky head spasms characteristic of cervical dystonia.

The findings from our study that superior collicular processing is disrupted in both patients and relatives harbouring the endophenotype (an abnormal TDT), supports the hypothesis that disrupted superior collicular processing is involved temporal discrimination. As an abnormal TDT is a mediational endophenotype for cervical dystonia, we might assume that disordered sensory processing in the superior colliculus is also involved in the pathogenesis of this condition.

### Potential Limitations

While the results of this study support a model of reduced superior collicular GABAergic activity as contributing to sensory processing abnormalities and motor features of cervical dystonia, our study cannot determine the exact level at which this deficit arises; this GABAergic deficit may, in fact, be upstream of the superior colliculus. Further research is required to determine this. A Dynamic Causal Modelling (DCM) study may be instructive in this regard as it may identify where the superior colliculus fits within a given brain network and how these connections are impacted by abnormal TDTs and dystonia.

## Methods

### Participants and Methods

Ethical approval for this work was granted by the Ethics and Medical Research Committee, St. Vincent’s University Hospital, Elm Park, Dublin 4, Ireland. All experiments were performed in accordance with relevant guidelines and regulations. Written informed consent was obtained from all participants for study participation and publication of identifying information/images.

### All participants

To examine any functional differences between those with a normal TDT versus those with an abnormal TDT the study was planned to have an equal representation from the following four groups; cervical dystonia patients, relatives with abnormal TDT, relatives with normal TDTs and healthy controls.

Sixty-four, age- and sex-matched participants (16 cervical dystonia patients, 16 first-degree relatives with abnormal TDT, 16 relatives with normal TDT and 16 healthy controls) were recruited. The mean age of study participants was 52.2 years (SD ± 7.75 years). Forty-two were women.

All participants had normal cognition, normal visual acuity, absence of sensory symptoms and a normal sensory examination.

### Cervical dystonia patients

Sixteen cervical dystonia patients (10 women; four familial, 12 sporadic) (mean age 53.5 years; SD ± 6.9 years) were recruited from the dystonia clinic at St. Vincent’s University Hospital. Each patient’s clinical diagnosis was confirmed by two neurologists with expertise in dystonia. Thirteen patients were receiving regular botulinum toxin injections for their dystonia at the time of scanning. The mean time since last injection in these patients was 41 days (SD ± 24 days).

Amongst the group receiving regular botulinum toxin injections, three patients were taking regular clonazepam and four patients were on anti-depressant medication. Of those not receiving regular botulinum toxin injections, none were taking regular medications.

### Unaffected relatives

Thirty-two unaffected first-degree relatives were recruited (22 women, mean age 52.1 years; SD ± 8.7 years). Seven had first-degree relatives with familial cervical dystonia; 25 had first-degree relatives with sporadic cervical dystonia. All were clinically examined by two neurologists with expertise in dystonia; none had any evidence of dystonia or dystonic tremor.

### Healthy control participants

From hospital staff and relatives of the research team sixteen healthy control participants were recruited (10 women; mean age 51.0 years; SD ± 8.0 years).

### Sensory testing

Visual and tactile TDT testing was carried out in a single session, in a sound-proofed, darkened room. This method and has been previously described (Kimmich *et al*., 2011).

### Functional Magnetic Resonance Imaging (fMRI)

#### Participants

Structural and functional MRI images were acquired from all 64 age- and sex-matched participants that participated in the behavioural experiment (16 cervical dystonia patients, 32 first-degree relatives and 16 healthy controls).

*Billington et. al* in a previous study investigated the processing of looming at whole brain and superior collicular level^41^. Their study was statistically powered to show differences between loom and recede in a cohort of ten participants aged between 20–40 years. As our study was conducted on an older population we increased the number of participants by 60% to ensure the study was sufficiently appropriately powered.

#### Structural acquisition

MRI data was collected on a Philips 3T Achieva MRI Scanner. A high-resolution three dimensional T1-weighted magnetisation-prepared rapid-acquisition gradient echo (MPRAGE) sequence was acquired (TR = 8.4 ms; TE = 3.9 ms, TI = 1150 ms, flip angle = 8 degrees) with a transverse orientation, a 256 × 256 matrix size and 0.9 mm isotropic voxels.

#### Functional acquisition

Functional images were collected using 40 slices covering the whole brain (slice thickness 3 mm, inter-slice distance 0 mm, in-plane resolution 3 × 3 mm) with an echo planar imaging (EPI) sequence (TR = 2 s, TE = 25 ms, flip angle = 90°). To ensure full coverage of the superior colliculi we orientated the slices parallel to the brainstem at the height of the pons. The first four volumes from each run were discarded to avoid T1 equilibrium effects.

#### fMRI stimulus presentation

**Figure 6:**
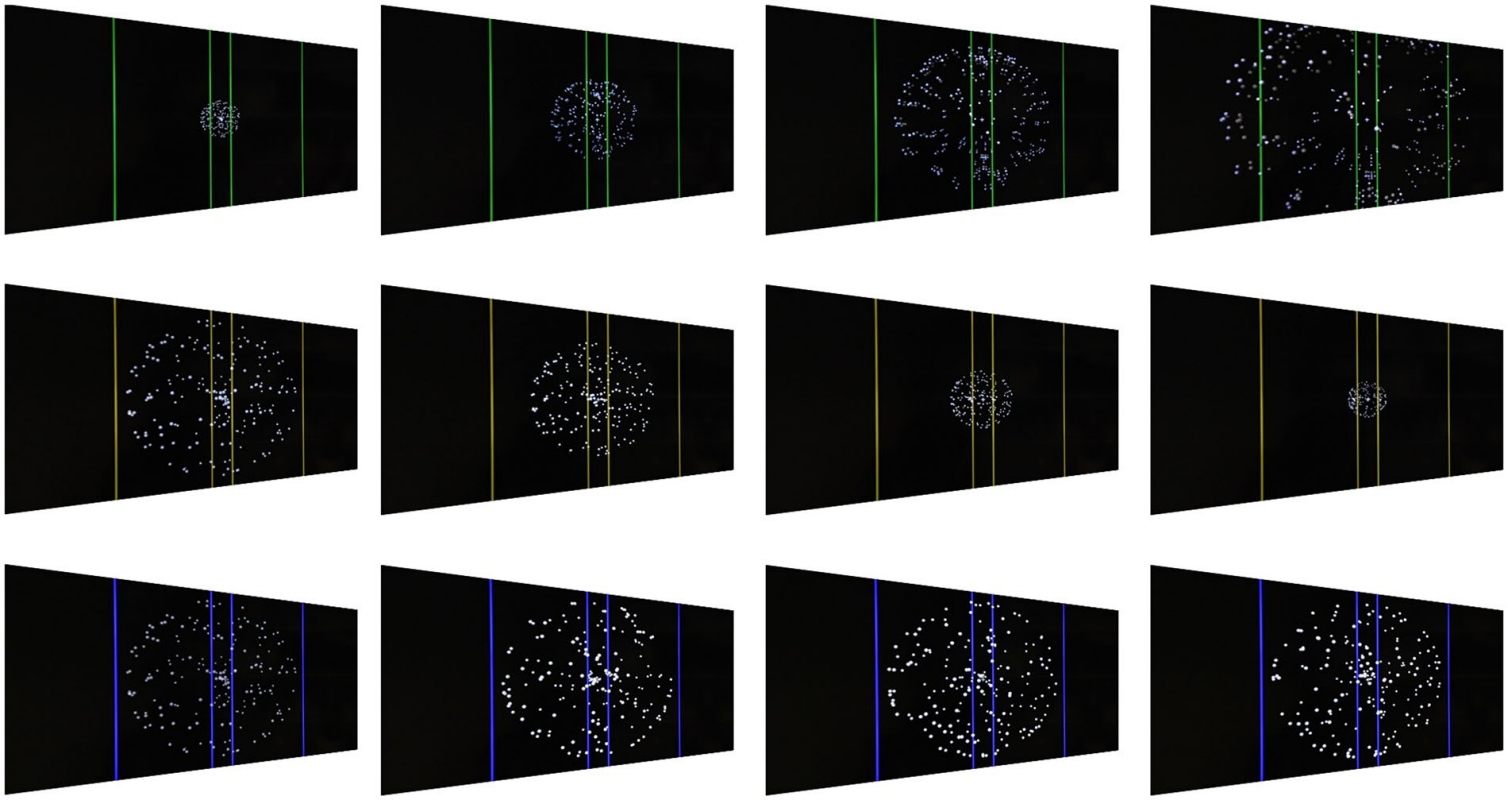
Experimental visual stimulus paradigm. Screen capture of the sequence of events that occurs during each of the three conditions incorporated into the experimental paradigm. The colour of the vertical lines indicates the trial type: loom (green), recede (yellow), random (blue). In the looming-motion condition, the size of the sphere expands during motion towards the outer vertical lines before disappearing. The receding-motion condition is the reverse of the looming-motion condition; the sphere begins at its maximum diameter and contracts towards the inner vertical lines before disappearing. The random-motion condition consists of an unchanging sphere volume with randomly moving points that maintain the same velocity of the previous conditions.

The visual paradigm, based on^41^ was an event-related design developed in Presentation Software (Neurobehavioral Systems Inc., Albany, CA, USA) and viewed by participants by way of an angled mirror located 30.48 cm from their eyes. To increase the perceived effect of 3-dimensional movement, thus increasing the likelihood of superior colliculus activation, a patch covered the right eye for the duration of the experiment. Participants were presented with three stimulus conditions: looming, receding and random motion (Fig. 6).

#### fMRI analysis

Statistical parametric mapping software (SPM12 www.fil.ion.ucl.ac.uk/spm), running under Matlab 8.5 (Mathworks, Sherborn, MA, USA), was used to pre-process and analyse the data according a previously described pipeline^59^. Briefly, pre-processing consisted of resetting the origin, realignment, unwrapping, co-registration, segmentation and normalisation.

The origin was reset to the anterior commissure to align functional and anatomical images in the same plane^60^. The first four images from each session were discarded to allow for equilibrium magnetisation. EPI blood oxygen level-dependent (BOLD) images were re-aligned and re-sliced using a six parameter spatial transformation with the first non-discarded image as a reference. Estimated motion parameters calculated during the re-alignment step were saved for later use as nuisance regressors in the first level general linear model. Co-registration of functional and structural T1-weighted images was completed automatically and confirmed by careful visual inspection to ensure accurate alignment. The unified segmentation routine was employed to perform the segmentation bias correction and spatial normalisation. Structural and functional volumes were normalised in Montreal Neurological Institute (MNI) space using the standard International Consortium for Brain Mapping (ICBM) template to facilitate group analysis. Smoothed images were generated using a kernel with 8 mm full-width at half maximum for whole brain analysis. Region of interest (ROI) analysis at the individual level was carried out on unsmoothed data. A single general linear model (GLM) was created for each subject which incorporated the regressors of interest (loom, recede and random motion) and nuisance regressors to account for discontinuity between recordings. The duration of each event corresponded to the timing of stimulus movement and was set to one second. The time-period between stimulus offset and the button press response was excluded from each event to avoid modelling an early motor preparatory response. The six movement parameters estimated during the re-alignment procedure were included as nuisance regressors to account for unwanted movement.

### Whole brain analysis

A whole brain one-sample and two-sample t-test using condition estimates (beta values) from a first level effect GLM analysis was performed to compare whole brain activation associated with each experimental condition (loom, recede, random motion). A set of contrast images for each participant was generated for testing at group level. The contrast images were divided into two groups: basic 1-sample t-test which tested for activation against a 0 background (condition>/<0) and contrast 2-sample t-tests which looked for differences in activation between different conditions (condition A>/<condition B). The contrast images were generated at the first level and then submitted to second level analysis. To test for group differences in superior colliculus activation the 64 participants were divided in to two groups: those with abnormal TDT (n = 32, patients and abnormal relatives) and normal TDT (n = 32, normal relatives and controls). A repeat 2^nd^ level analysis was calculated for each group incorporating the contrasts examined in the whole group analysis.

### Superior colliculus boundary definition for region of interest analysis

**Figure 7:**
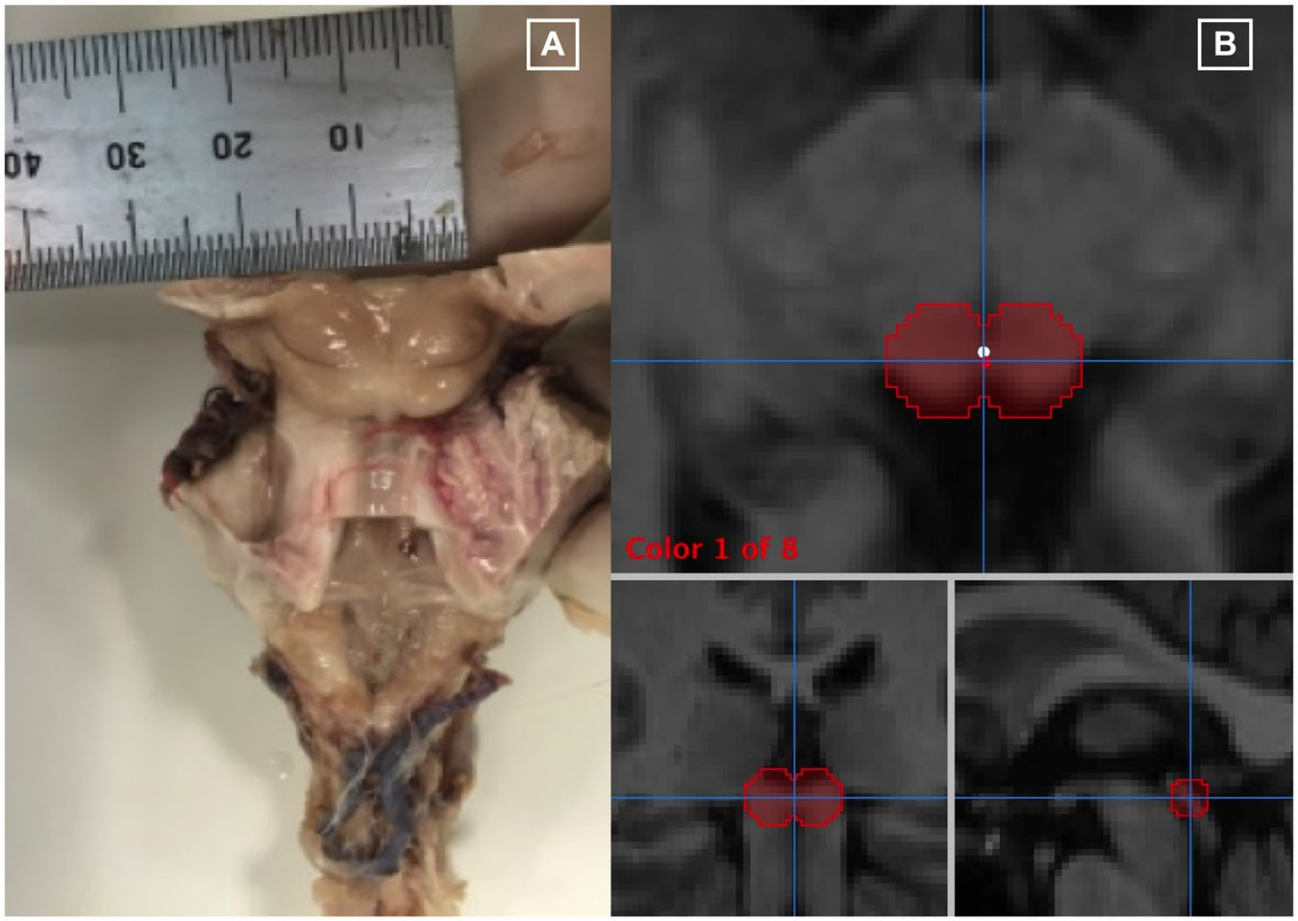
Region of interest anatomical definition: (**A**) Human cadaver brainstem specimen used during a neuro-pathological dissection. A consultant neuropathologist dissected the brainstem specimen and identified the anatomical landmarks of the left and right superior colliculi. The diameter of each superior colliculus was measured and noted to be 7 mm. (**B**) Superior collicular 3-D regions of interest on T1-weighted structural brain images. Three radiological views are shown; axial, coronal and sagittal respectively. Mango image processing software (Lancaster, Martinez; www.ric.uthscsa.edu/mango) was used to create the 3-D regions of interest using pre-defined anatomical boundaries from the neuro-pathological dissection.

Imaging of the superior colliculus is difficult due its small size, its proximity to major blood vessels and anatomical variation that exists between subjects^61^. Accurate delineation of superior collicular anatomical boundaries is necessary to ensure optimal region of interest analysis^45^. To achieve this, we identified the anatomical landmarks of the superior colliculus during neuropathological dissection of human brainstem specimens. A consultant neuropathologist dissected the brainstem specimen and identified the anatomical boundaries of left and right superior colliculus (Fig. 7A). The diameter of each superior colliculus was measured and noted to be 7 mm. Using these pre-defined anatomical boundaries, we created 3-D regions of interest (ROIs) using Mango image processing software (Lancaster, Martinez; www.ric.uthscsa.edu/mango). Spheres were hand-placed on individual participant’s superior colliculi by a neuroradiologist blinded to participant status (Fig. 7B).

Structural T1-weighted images were used for ROI definition. The axial-plane centre point was defined as a point equidistant from the posterior surface of the superior colliculus to the anterior border of the superior colliculus (posterior border of the posterior commissure). On the sagittal plane a vertical line was drawn from the superior border of the superior colliculus to the indentation between the superior and inferior colliculi: where this line crossed a line equidistant to the medial and lateral border of the superior colliculus our centre-point was set. The measured diameter of an individual superior colliculus at gross dissection was 7 mm. To compensate for the difference between slice-thickness (3 mm) and inter-slice difference (0 mm), 3 mm was added to the diameter prior to estimating the radius.

$$7\,{\rm{mm}}\,({\rm{measured}}\,{\rm{diameter}})+3\,{\rm{mm}}\,({\rm{difference}})=10\,{\rm{mms}}\div{\rm{2}}={\rm{5}}\,{\rm{mm}}\,({\rm{radius}})$$

Individual ROIs were then incorporated into the pre-processing pipeline.

### Region of interest analysis

#### Effect of condition

The raw event-related time courses were extracted from each participant’s superior colliculus using methodology from a previous study in healthy control adult participants^41^. The three stimulus types (looming, receding and random motion) were modelled at the first level and incorporated into a second level analysis. To calculate the change in BOLD signal over time within the region of interest, percent signal change time courses were estimated for individual subjects. The peak was taken as a more accurate estimate of the magnitude of an individual’s functional activation than the beta estimate^62^. Participants were grouped according to normal or abnormal TDT for ROI analysis. The extracted peak percent signal change was directly compared by means of an independent Student *t*-test for three main contrasts - loom > random, recede > random and loom > recede. By comparing contrast of conditions (as opposed to conditions) within subjects, we reduced the potential for noise that may arise from inter-subject variability.

#### Correlation analysis with temporal discrimination threshold

To explore the relationship between temporal discrimination and BOLD activations at superior collicular level, participant TDT Z-scores was correlated with the peak percentage signal change in the region of interest for the three main contrasts (looming > random, recede > random, loom > recede). Participants were grouped according to normal or abnormal TDT for correlation analysis.

### Data availability

All data generated or analysed during this study are included in this published article^63^.
